# Regulation of the Wnt signaling pathway during myogenesis by the mammalian SWI/SNF ATPase BRG1

**DOI:** 10.3389/fcell.2023.1160227

**Published:** 2023-07-07

**Authors:** Tapan Sharma, Monserrat Olea-Flores, Anthony N. Imbalzano

**Affiliations:** Department of Biochemistry and Molecular Biotechnology, UMass Chan Medical School, Worcester, MA, United States

**Keywords:** Wnt signaling, myogenesis, chromatin remodeling enzymes, BRG1, BRM, SWI/SNF, β-catenin, bromodomain

## Abstract

Skeletal muscle differentiation is a tightly regulated process, and the importance of the mammalian SWI/SNF (mSWI/SNF) chromatin remodeling family for regulation of genes involved in skeletal myogenesis is well-established. Our prior work showed that bromodomains of mSWI/SNF ATPases BRG1 and BRM contribute to myogenesis by facilitating the binding of mSWI/SNF enzymes to regulatory regions of myogenic and other target genes. Here, we report that pathway analyses of differentially expressed genes from that study identified an additional role for mSWI/SNF enzymes via the regulation of the Wnt signaling pathway. The Wnt pathway has been previously shown to be important for skeletal muscle development. To investigate the importance of mSWI/SNF enzymes for the regulation of the Wnt pathway, individual and dual knockdowns were performed for BRG1 and BRM followed by RNA-sequencing. The results show that BRG1, but not BRM, is a regulator of Wnt pathway components and downstream genes. Reactivation of Wnt pathway by stabilization of β-catenin could rescue the defect in myogenic gene expression and differentiation due to BRG1 knockdown or bromodomain inhibition using a specific small molecule inhibitor, PFI-3. These results demonstrate that BRG1 is required upstream of β-catenin function. Chromatin immunoprecipitation of BRG1, BRM and β-catenin at promoters of Wnt pathway component genes showed binding of BRG1 and β-catenin, which provides further mechanistic insight to the transcriptional regulation of these genes.

## Introduction

The skeletal muscle system is the largest organ of the muscular system, comprising about 30%–40% of the body mass. Skeletal muscles are responsible for voluntary body movements in coordination with the skeletal and nervous systems. These muscles originate from stem cells in the mesodermal layer during embryonic development (Stern et al., 1995; Buckingham et al., 2003; Braun and Gautel, 2011). In adults, muscle stem cells known as satellite cells are quiescent until they encounter a physical insult or injury (Motohashi and Asakura, 2014). Upon injury, satellite cells undergo rapid proliferation to generate mononuclear myocytes that increase expression of muscle-specific transcription factors MyoD and Myogenin and concurrently withdrawal from the cell cycle (Tajbakhsh et al., 1998; Roy et al., 2002; Bentzinger et al., 2012). This is followed by fusion of mononuclear myocytes into differentiated multinuclear myotubes. Due to its enormous capacity to regenerate, skeletal muscle is well-characterized as a model for studying tissue development and cellular differentiation. A better understanding of this process is key to developing therapies for muscular disorders like dystrophies, rhabdomyosarcoma, and myositis.

The SWI/SNF enzymes are the largest family of ATP-dependent chromatin remodelers in eukaryotes, and their roles in transcriptional regulation have been well-documented (Müller and Leutz, 2001; Clapier and Cairns, 2009; Laengst et al., 2015). Mutations in many of these complex subunit proteins are associated with cancer (Weissman and Knudsen, 2009; Romero and Sanchez-Cespedes, 2014). Mammalian SWI/SNF (mSWI/SNF) remodelers exist as multi-subunit complexes that can assemble combinatorially, with different complexes assuming specific roles (Wang et al., 1996). Since chromatin remodeling is an ATP-dependent process, the ATPase subunit provides functional relevance to the complex. Brahma (BRM) and Brahma-Related Gene 1 (BRG1) are mutually exclusive ATPases found in all SWI/SNF complexes within a mammalian cell. Loss of enzymatic activity of the BRG1 ATPase subunit or mutations in the gene encoding it has been previously shown to be embryonic lethal (Bultman et al., 2000). Previous work from our lab and others has shown the importance of BRG1 and BRM in skeletal muscle development (de la Serna et al., 2001; Simone et al., 2004; de la Serna et al., 2005; De La Serna et al., 2006; Ohkawa et al., 2006; Ohkawa et al., 2007; Forcales et al., 2012; Comai and Tajbakhsh, 2014; Albini et al., 2015; Chal and Pourquié, 2017).

mSWI/SNF complexes are also known to regulate signaling pathways in a tissue-specific manner by coordinating gene activation with help from signature transcription factors. Simone et al. had shown that, in skeletal muscle, p38/MAP kinase activity is required for phosphorylation of the BAF60c subunit, which promotes recruitment of mSWI/SNF (Simone et al., 2004). In the absence of p38 kinase activity, muscle-specific transcription factors MyoD and MEF2 bind to histone acetyltransferases around regulatory regions but cannot activate gene expression due to lack of mSWI/SNF engagement in this process. Similarly, the GATA-1 transcription factor can interact with the mSWI/SNF complex to activate β-globin gene expression during myeloid differentiation (Kadam et al., 2000). Analogous interactions between tissue-specific transcription factors and mSWI/SNF remodelers have been reported in case of neuronal (Seo et al., 2005; Eroglu et al., 2006), cardiac (Lickert et al., 2004; Zhou et al., 2009; Lei et al., 2013), adipocyte (Kadam and Emerson, 2003; Debril et al., 2004; Salma et al., 2004), hepatocyte (Gresh et al., 2005), and retinal (Das et al., 2007) differentiation.

Skeletal muscle differentiation is regulated by precisely coordinated signaling mechanisms triggered by mechanical and chemical stimuli, each of which can activate downstream signaling pathways (Olson, 1993; Chang, 2007; Perdiguero et al., 2009; Moreira-Pais et al., 2020). Some of the most well-studied signaling pathways in skeletal muscle include the p38 MAPK (Simone et al., 2004; Serra et al., 2007), IGF1 (Chang, 2007; Serra et al., 2007), PI3K/AKT/mTOR (Bassel-Duby and Olson, 2006), NFκB (Chang, 2007), Wnt (Moreira-Pais et al., 2020), JAK/STAT (Yin et al., 2013), and Ca^2+^/Calmodulin-dependent pathways (Olson, 1993; Chang, 2007). Canonical and non-canonical Wnt signaling has been shown to play differential roles in myoblast proliferation and skeletal muscle differentiation at early and terminal stages (Münsterberg et al., 1995; Tajbakhsh et al., 1998; Miller et al., 1999; Petropoulos and Skerjanc, 2002; Eliazer et al., 2019). Most of these signaling pathways can crosstalk with each other, and therefore an understanding of their regulation and integration is key to understanding development and regeneration, as well as to development of therapeutics that can cure skeletal disorders.

The canonical Wnt signaling pathway is dependent on cellular β-catenin levels (Miller et al., 1999; Petropoulos and Skerjanc, 2002; Dennis and Bradshaw, 2003; Otto et al., 2008). In the absence of a Wnt ligand-bound frizzled (Fzd) receptor, a downstream β-catenin degradation protein complex is active (Aberle et al., 1997; Petropoulos and Skerjanc, 2002). GSK3β is a component kinase of this complex that phosphorylates β-catenin and marks it for subsequent ubiquitination and proteasomal degradation (Aberle et al., 1997). In the presence of a Wnt ligand, the β-catenin degradation protein complex is tethered to the frizzled receptors in the membrane with the help of disheveled (Dvl) proteins, which blocks phosphorylation of β-catenin and promotes its stabilization (Otto et al., 2008). Stable β-catenin translocates to the nucleus and associates with transcription factors from the TCF/LEF/TLE families to regulate downstream target expression (Wagner et al., 2000). *In vitro* studies have utilized pharmacological inhibition of GSK3β to stabilize cellular β-catenin levels and mimic Wnt activation (Chang, 2007).

The role of Wnt signaling in muscle was identified about 30 years ago (Münsterberg et al., 1995; Stern et al., 1995). It is now known that Wnt signaling can contribute to all stages of skeletal muscle development (Moreira-Pais et al., 2020), including satellite cell proliferation (Otto et al., 2008), niche renewal (Yin et al., 2013; Eliazer et al., 2019), onset of differentiation by regulating MRF expression (Tajbakhsh et al., 1998), myocyte fusion (Rochat et al., 2004; Pansters et al., 2011) and terminal differentiation (Brack et al., 2007; Brack et al., 2008). Wnt and Notch signaling work antagonistically to regulate early and later stages of differentiation (Brack et al., 2008). Notch signaling is required for early activation and expansion of the satellite cell pool while Wnt signaling is responsible for fusion of these activated progenitors to form myotubes/myofibers (Brack et al., 2008).

A recent study from our group demonstrated that the bromodomains of the BRG1 and BRM ATPases are crucial for timely exit of myoblasts from the cell cycle and formation of well-developed myotubes *in vitro* and *in vivo* (Sharma et al., 2021). Here, we present new bioinformatic analysis of the genes differentially expressed in the presence of the inhibitor of the BRG1 and BRM bromodomains that identifies affected cell signaling pathways. Most notably, the Wnt signaling pathway was dependent on BRG1 and BRM bromodomains. siRNA mediated perturbation of BRG1 and BRM expression followed by Panther pathway analysis determined a key dependence of the Wnt signaling pathway on BRG1 but not on BRM. Chromatin IP assays showed binding of BRG1 on gene promoters of Wnt component genes as a function of myoblast differentiation, thus providing a mechanism of BRG1-dependent regulation of the Wnt signaling pathway. This work shows that mSWI/SNF enzymes regulate the Wnt signaling pathway in addition to regulating myogenic genes and genes controlling cell cycle, thereby expanding our understanding of the mSWI/SNF function during skeletal muscle differentiation.

## Materials and methods

### Antibodies and chemicals

Primary antibodies used for WB against BRG1 (sc-17796; 1:1,000), LaminB1 (sc-56144; 1:10,000) and β-catenin (sc-7963, 1:1,000) were purchased from Santa Cruz Biotech, United States. Myosin Heavy Chain (MHC) (Catalog# MF20; 1:1,000 for WB; 1:100 for ICC) was purchased from the Developmental Studies Hybridoma Bank, University of Iowa, United States. Antibodies used for chromatin IPs against β-catenin (Cell Signaling Technology, Inc., United States, Catalog# 8,480, 2 μL per μg of chromatin), BRG1 (Santa Cruz Biotech, United States, sc-17796, G-7X, 3–4 μL per μg of chromatin) and BRM antisera (3–4 μL per μg of chromatin) have been previously described (de la Serna et al., 2005; Sharma et al., 2021). PFI-3 (catalog# 15267) and CHIR99021 (catalog# 13122) were purchased from Cayman Chemicals, United States. Lysis buffers for ChIP assays were purchased from Cell Signaling Technology, United States (SimpleChIP^®^ Enzymatic Cell Lysis Buffers A & B, 14,282; SimpleChIP^®^ Chromatin IP Buffers, 14,231). Dulbecco’s modified Eagle’s medium (DMEM) was purchased from ThermoFisher Scientific (#11965118). Vectastain elite ABC (PK-6200) and HRP DAB substrate (SK-4100) kits were purchased from Vector Laboratories, United States.

### Cell culture

C2C12 cells were maintained and differentiated as previously described (Sharma et al., 2021). PFI-3 was added to cell culture media at a final concentration of 50 μM for all experiments. For Wnt pathway rescue experiments, 2.5 μM of the GSK3β inhibitor CHIR99021, 10 mM LiCl, 1% DMSO, or 10 mM NaCl were added at the time of induction of differentiation.

### siRNA transfection

siRNA oligos against BRG1 (siGENOME mouse Smarca4 pool #M-041135-01-0020, 50nM; siGENOME mouse Smarca4 #D-041135-03-0050, 25nM, referred to as siBRG1-A; and siGENOME mouse Smarca4 #D-041135-04-0050, 25nM, referred to as siBRG1-B) and BRM (siGENOME mouse Smarca2 pool #M-056591-00-0020, 50 nM) and non-targeting control (SMARTpool ON-TARGETplus scrambled # D-001810-10–20, 50 nM) were purchased from Dharmacon Horizon Discovery Ltd., United States. C2C12 cells were transfected as previously described (Sharma et al., 2021) using indicated concentrations of different siRNAs and harvested at indicated times for further analysis.

### Immunofluorescence

C2C12 cells were stained and analyzed for immunofluorescence as previously described (Sharma et al., 2021). Immunostaining was performed with MHC antibody (dilution in antibodies section) and DAPI for nuclear staining. Fusion index was calculated as the percentage of nuclei/cells stained with myosin heavy chain as compared to total number of nuclei/cells (Metzinger et al., 1993).

### Immunoblot analysis

C2C12 cells after indicated treatments were washed with PBS twice and were scraped using a cell lifter into 1 mL PBS. The resulting cell suspension was collected, and cells were pelleted by centrifugation. The pellets were lysed in 500 μL RIPA buffer (50 mM Tris-HCl, pH7.4, 150 mM NaCl, 1 mM EDTA, 1% NP-40% and 0.25% sodium deoxycholate) supplemented with 1X protease inhibitor cocktail (Sigma Aldrich, P8340). The lysates were incubated on a rocker for 15 min at 4 °C to allow lysis of cell membranes, followed by sonication in an ultrasonic bath sonicator for 3 cycles of 30 s on/off. Samples were centrifuged at 14,000 *g* for 10 min at 4 °C and supernatants were collected. Protein concentrations were determined using a Pierce™ BCA protein assay kit (ThermoFisher Scientific, United States) according to the manufacturer’s protocol. 20–50 μg of total protein with 5X loading dye (5% β-Mercaptoethanol, 0.02% Bromophenol blue, 30% Glycerol, 10% SDS, 250 mM Tris-Cl, pH 6.8) was boiled at 95 °C for 10 min and subjected to 8% SDS-polyacrylamide gel electrophoresis followed by electroblotting onto Immobilon-P PVDF membranes (Merck Millipore, United States). The membranes were then blocked using 5% non-fat milk in PBS for 30 min followed by overnight incubation at 4 °C with primary antibody against protein of interest at the desired dilution in 2% non-fat milk prepared in PBS or TBS. This was followed by 3 washes with TBS containing 0.1% Tween-20 for 5 min each at room temperature. The membranes were then incubated with HRP-conjugated anti-mouse or anti-rabbit secondary antibodies (1:2500, GE Healthcare Life Sciences) diluted in 2% non-fat milk prepared in PBS for 1 h at RT followed by 3 washes with TBS containing 0.1% Tween-20 for 5 min each at room temperature. Immuno-reactive bands were visualized by chemiluminescence with ECL Plus (GE Healthcare Life Sciences) using an Amersham Imager 600 (GE Healthcare Life Sciences). ImageJ software (NIH) (Schneider et al., 2012) was used to calculate band intensities from 3 independent experiments.

### Cell fractionation and Co-Immunoprecipitation

Cells grown on 10 cm dish were treated according to the relevant treatment group. On the day of harvest, culture media was aspirated, and cells were washed with ice-cold PBS twice to wash off residual media. Cells were scraped, resuspended in 1 mL ice-cold PBS, and collected in 1.5 mL Eppendorf tubes. Cells were pelleted at 2500 rpm for 5 min and the cell pellet was resuspended in 300ul of hypotonic buffer (10 mM HEPES, pH 7.9, with 1.5 mM MgCl2 and 10 mM KCl with freshly added protease inhibitor (Sigma Aldrich, P8340)). After 10 min of incubation on ice, with gentle vortexing 2-3 times in between, the cell suspension was centrifuged at 10,000 rpm for 1 min at 4 °C. The supernatant comprises the cytosolic lysate and was collected in a fresh Eppendorf tube. The nuclei in the pellet were washed gently with 1 mL hypotonic buffer and then resuspended using 300ul of RIPA buffer (50 mM Tris-HCl, pH7.4, 150 mM NaCl, 1 mM EDTA, 1% NP-40% and 0.25% sodium deoxycholate, add protease inhibitor (Sigma Aldrich, P8340) fresh). The suspension was incubated on ice for 10–15 min followed by lysis using 27G/28G syringe needles. The samples were centrifuged at 10000rpm for 5 min at 4 °C and supernatant (nuclear lysate) was used for co-immunoprecipitation experiments.

To set up co-IP, protein concentrations of the lysates were determined using Pierce™ BCA protein assay kit (ThermoFisher Scientific, United States) according to the manufacturer’s protocol. Equal amounts of protein was aliquoted to fresh pre-cooled Eppendorf tubes. Relevant antibodies for desired proteins were added and reactions were incubated overnight with rotation at 4 °C. Next morning, 40ul of equilibrated protein A beads (Catalog#10001D, ThermoFisher Scientific, United States) were added to each IP mix and incubated with rotation for additional 1–2 h. The beads were washed with 500ul cold RIPA lysis buffer thrice to remove unbound proteins. The pulled down protein-Ab complex was eluted in 50ul of RIPA buffer by incubating the beads at RT for 2 h. The eluant was collected and mixed with 5X loading dye (5% β-Mercaptoethanol, 0.02% Bromophenol blue, 30% Glycerol, 10% SDS, 250 mM Tris-Cl, pH 6.8) and electrophoresed using 8% SDS-polyacrylamide gels.

### RNA isolation and quantitative RT-PCR

RNA was extracted using Trizol (ThermoFisher) as per the manufacturer’s protocol. cDNA was prepared using 2 μg of total RNA quantified using a Nanodrop1000 spectrophotometer (ThermoFisher Scientific) using Superscript III First Strand Synthesis Kit (Invitrogen) according to manufacturer’s protocol. At least three independent biological replicates for qRT-PCR were performed with technical duplicates for each sample using 1 μL each of forward and reverse primers (10 μM stocks), 1 μL cDNA, 5 μL of Fast SYBR Green 2X Master Mix (Applied Biosystems), and final reaction volume was adjusted to 10 μL per reaction. All qPCRs were run in QuantStudio 3 RT-PCR machine (Applied Biosystems). ΔCT was calculated by subtracting the CT value for housekeeping gene (*Eef1α1*) from that of the target gene. ΔΔCT of each target gene was then calculated by subtracting the average of the ΔCT obtained in the control samples from ΔCT for each test sample. Relative gene expression was calculated using the 2^−ΔΔCT^ method (Livak and Schmittgen, 2001). Primers were purchased from Integrated DNA Technologies, Inc., United States. Primer sequences are listed in Supplementary Table S1.

### RNA-sequencing analysis

For RNA sequencing, total RNA samples in duplicate for each condition were evaluated for quality and concentration at the UMass Chan Medical School MBCL Fragment Analyzer services. Samples with RIN≥7 and 28S/18S ≥ 1.0 were sent to BGI Americas Corporation for library preparation and RNA sequencing (Huang et al., 2017). Libraries were sequenced and filtered for adapter-removal to generate clean fasta files. Reads were aligned to mm10 reference transcriptome using HISAT2. Gene expression levels were calculated using featureCounts (Liao et al., 2014). DEseq2 (Love et al., 2014) and EdgeR (Robinson et al., 2010) algorithms were used to detect differentially expressed genes (DEG). Transcripts with log2fold change ≥ ±0.5 and p-adjust <0.05 were identified as significantly dysregulated and were considered for further analysis. Pathway analysis was performed using the Panther database (Thomas et al., 2003; Mi et al., 2013). Volcano plots, dotplots and heatmaps were generated using ggplot2 (Wickham, 2016), Cluster profiler (Wu et al., 2021) and pheatmap (Pheatmap, 2019) packages in RStudio.

### Chromatin immunoprecipitation assay

Chromatin immunoprecipitation assays were performed as described previously (Witwicka et al., 2019). Quantification was performed using the fold enrichment method (2^-(Ct sample–Ct IgG)^) and shown as relative to a control region from a gene desert on mouse chromosome 6 as performed previously (Sharma et al., 2021). Sequences of primers used for ChIP assays are listed in Supplementary Table S1.

### Statistical analysis

Data generated from qRT-PCR, western blots, immunofluorescence, and chromatin immunoprecipitation assays are shown as mean ± the standard deviation of at least three independent biological replicates. Statistical analyses were performed using paired Student’s t-test in Prism9 (Graphpad Prism Software Inc., Unite States). Significance is displayed with *p < 0.05, **p < 0.01 and ***p < 0.005.

## Results

### Inhibition of BRG1 and BRM bromodomains affects the Wnt signaling pathway

**FIGURE 1:**
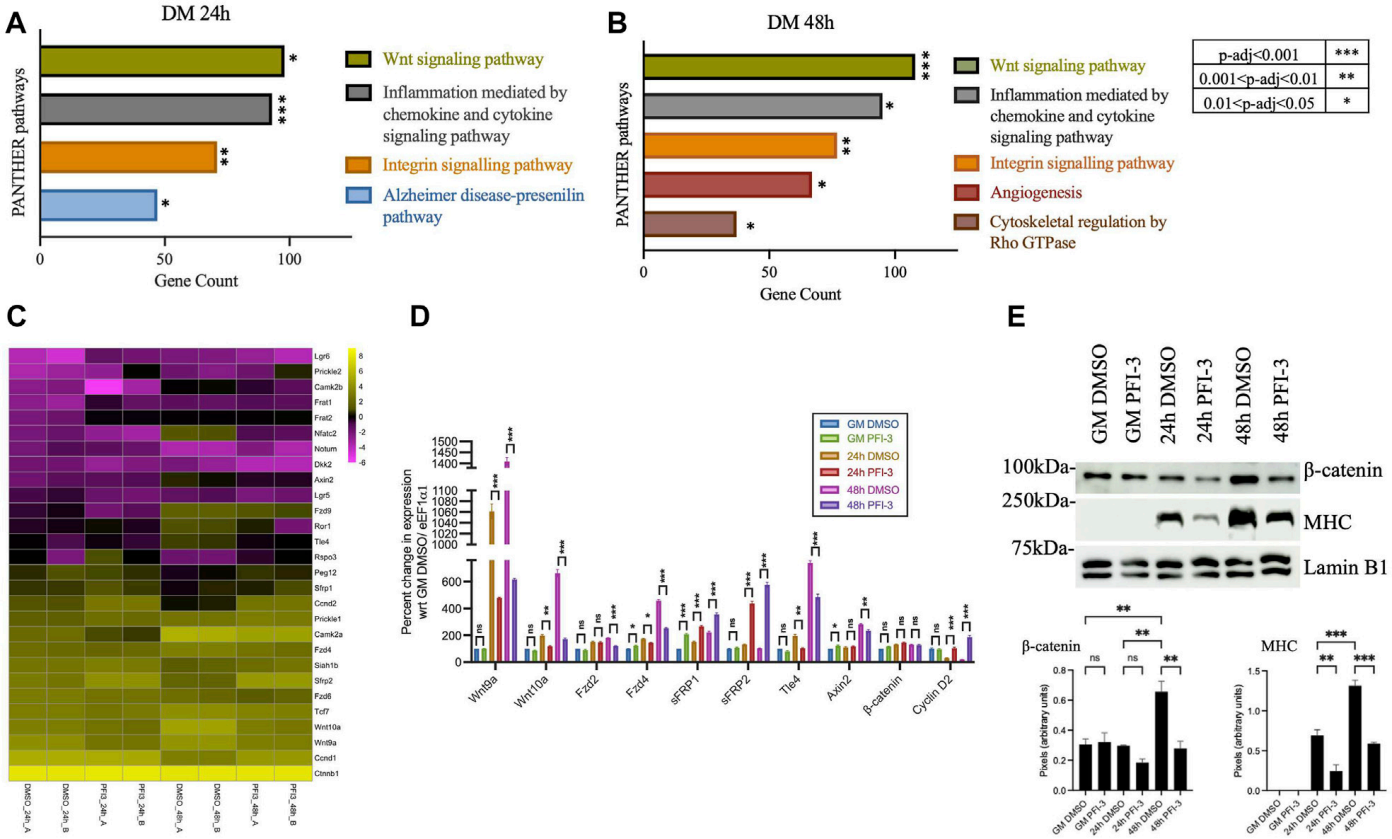
Inhibition of BRG1 and BRM bromodomains affects the Wnt signaling pathway. PANTHER pathway analysis of differentially expressed genes identified from previously published (Sharma et al., 2021) RNA-sequencing analysis of PFI-3 treated C2C12 myotubes differentiated for **(A)** 24 h and **(B)** 48 h showing the top significantly affected pathways, with *p*-value significance as shown in the key to the right of panel **(B)**. **(C)** Heatmap showing log_2_FPKM values for Wnt pathway-related genes from RNA-sequencing analyses in panels **(A)** and **(B)**. Data from the two independent replicates are shown side-by-side. **(D)** mRNA expression levels were determined by quantitative RT-PCR for Wnt-related genes- Ligands (*Wnt9a*, *Wnt10a*); Receptors (*Fzd2*, *Fzd4*); Antagonists (*sFRP1*, *sFRP2*); transcription factors (*Ctnnb1*/β-catenin, *Tle4*); downstream targets (*Axin2*, *Ccnd2*/Cyclin D2) from C2C12 myoblasts treated with DMSO or 50 μM PFI-3 for the indicated timepoints. Expression was normalized to control gene (*Eef1α1*) and change in expression is presented as percent change with respect to proliferation stage (GM) samples treated with DMSO, which were set at 100%. **(E)** Upper panel: Representative western blots for expression of β-catenin, myosin heavy chain (MHC) and Vinculin (loading control) at the indicated timepoints in C2C12 myoblasts treated with DMSO or PFI-3. Lower panel: Quantification of western blots for myogenin and myosin heavy chain. For panels **(D)** and **(E)**, the data represent the mean ± standard deviation from three independent experiments. ns, not significant, *p < 0.05, **p < 0.01, and ***p < 0.005 by Student’s t-test. GM, proliferating cells in growth media; DM; differentiating cells in differentiation media.

We recently reported that the bromodomains of mSWI/SNF ATPases BRG1 and BRM are crucial for regulation of myoblast differentiation in culture and skeletal muscle regeneration following injury *in vivo* (Sharma et al., 2021). Inhibition of bromodomain function using the selective inhibitor PFI-3 resulted in decreased expression of myogenic genes and an increase in the number of cells that failed to exit from cell cycle under differentiation conditions due to mis-regulation of cell cycle regulatory protein levels (Sharma et al., 2021). Mechanistically, bromodomain function was required for recruitment of the mSWI/SNF ATPases to regulatory regions of cell cycle-related and muscle-specific genes. In the present study, all differentially expressed genes (DEGs) from PFI-3 treated differentiating C2C12 myoblasts were analyzed using PANTHER (Thomas et al., 2003; Mi et al., 2013) to identify pathways regulated by BRG1 and BRM bromodomains. Transcriptional output related to the Wnt signaling pathway was the most significant alteration as measured by the number of affected genes at both 24h and 48 h post-differentiation (Figures 1A,B). All significantly altered pathways are shown. Other signaling pathways like integrin signaling and cytoskeletal regulation by Rho GTPase were also identified (Figures 1A,B). Both of these pathways are extra-cellular matrix (ECM) signaling related pathways and are known to be regulated by the mSWI/SNF family of chromatin remodelers (Olson, 1993; Egerman and Glass, 2014).

Volcano plots of all identified DEGs from 24 h to 48 h differentiated C2C12 myoblasts were generated, and some of the identified myogenic, cell cycle-related and Wnt-associated genes were labeled (Supplementary Figure S1). Wnt pathway component genes are highlighted by arrowheads. Inhibition of bromodomain function using PFI-3 did not alter the expression levels of *Brg1* or *Brm* transcripts (Supplementary Figure S1), as shown previously (Sharma et al., 2021). A heatmap of gene expression changes for differentially expressed Wnt pathway genes 48 h post-differentiation is shown in Figure 1C. Individual biological replicates are shown side-by-side. The dysregulated transcripts included those encoding the ligands Wnt9 and Wnt 11; frizzled membrane receptors Fzd1, Fzd2, Fzd4, and Fzd6; antagonists, sFRP1 and 2, Dkk2; R-spondin rspo3; transcription factors Tcf7, Tcf7l1, and Tle4, and others. Wnt target genes like those encoding cyclin D isoforms were also dysregulated. Expression of some of these Wnt component and target genes, along with other genes linked to Wnt signaling, were validated using qRT-PCR analysis of transcript levels in C2C12 myoblasts differentiated for 24 h and 48 h in the presence of PFI-3 (Figure 1D). The Wnt signaling pathway controls downstream target expression through β-catenin interacting with transcription factors belonging to the TCF/LEF/TLE families. Consistent with our findings, our previously published HOMER-based motif search of promoters of DEGs resulting from PFI-3 inhibition in differentiating C2C12 myoblasts identified the motif associated with TCF12 as one of the top 10 hits (Sharma et al., 2021).

Western blots indicate an increase in the β-catenin levels in differentiating cells compared to proliferating myoblasts and demonstrate that bromodomain inhibition decreases β-catenin protein expression levels (Figure 1E). Interestingly, β-catenin expression at the transcript level is unchanged due to PFI-3-induced bromodomain inhibition (Figures 1C,D), indicating regulation at the post-transcriptional level. These results show that inhibition of BRG1 and BRM bromodomains negatively impacts the transcription of genes belonging to the Wnt signaling network and post-translationally affects the levels of β-catenin.

### Knockdown of BRG1, but not BRM, affects the Wnt signaling pathway

**FIGURE 2:**
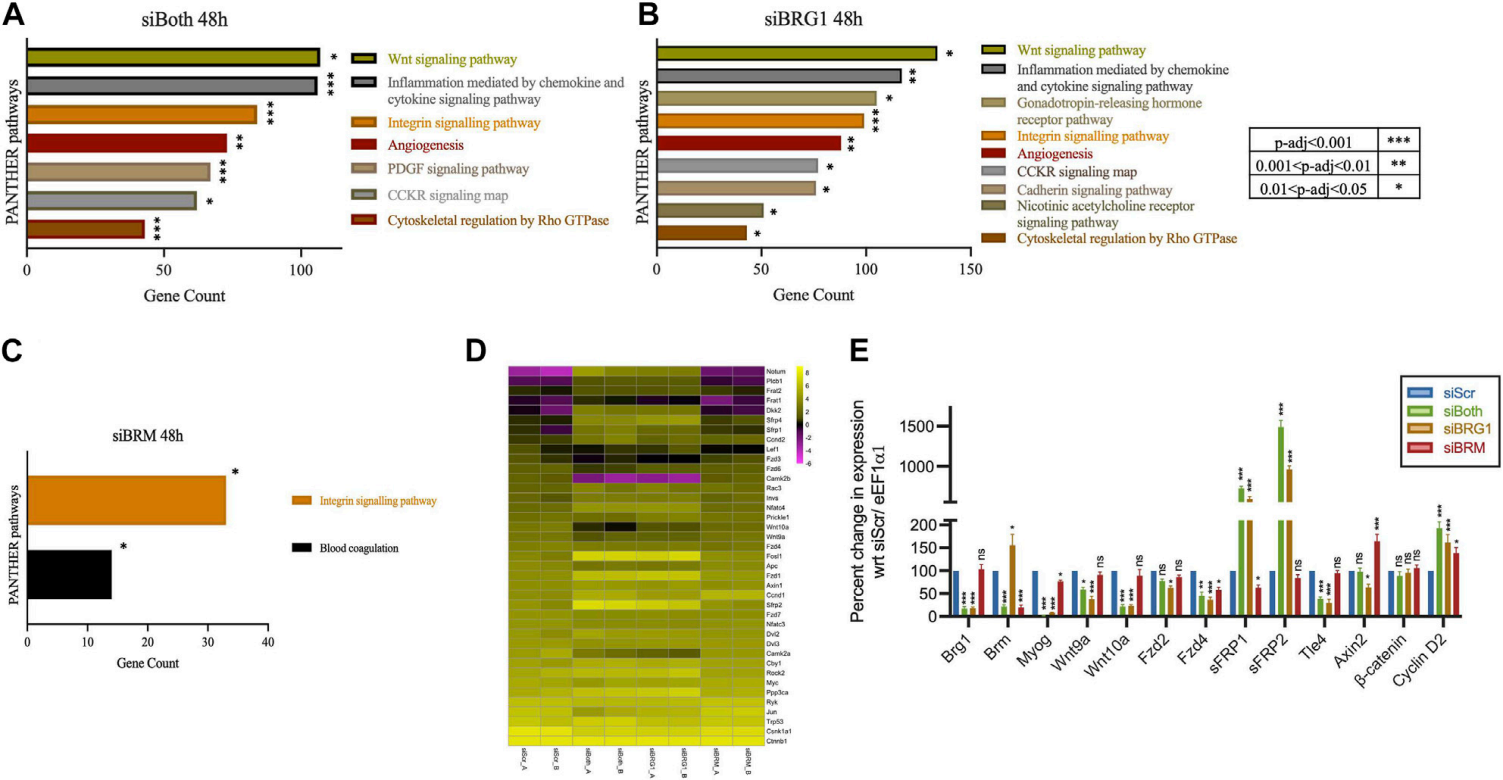
Knockdown of BRG1, but not BRM, affects the Wnt signaling pathway. PANTHER pathway analysis of differentially expressed genes identified from RNA-sequencing analysis of C2C12 myotubes differentiated for 48 h while being knocked down for **(A)** both BRG1 and BRM or **(B)** BRG1 only, as compared to non-targeting scrambled siRNA or **(C)** BRM only, as compared to non-targeting scrambled siRNA. Analyses show the top affected signaling pathways with *p*-value significance as shown in the adjoining key located to the right of panel **(B)**. **(D)** Heatmap showing log_2_FPKM values for Wnt pathway-related genes from RNA sequencing analysis of 48 h differentiated C2C12 myotubes that were treated with siRNA against non-targeting scrambled (siScr), both BRG1 and BRM (siBoth), BRG1 only (siBRG1), or BRM only (siBRM). Data from the two independent replicates are shown side-by-side. **(E)** mRNA expression data from qRT-PCR analysis of BRG1, BRM, Myogenin (*Myog*) and **(D)** mRNA expression data from qRT-PCR analysis of other Wnt related genes - Ligands (*Wnt9a*, *Wnt10a*); Receptors (*Fzd2*, *Fzd4*); Antagonists (*sFRP1*, *sFRP2*); transcription factors (*Ctnnb1*/β-catenin, *Tle4*); downstream targets (*Axin2*, *Ccnd2*/Cyclin D2) from 48 h differentiated C2C12 myotubes knocked down for BRG1 and/or BRM as described in **(D)**. Expression was normalized to a control gene (*Eef1α1*) and change in expression is presented as percent change with respect to siScr, which was set at 100%. Data represent the mean ± standard deviation from three independent experiments. ns, not significant, *p < 0.05, **p < 0.01, and ***p < 0.005 by Student’s t-test.

PFI-3 is a selective inhibitor of the bromodomains of BRG1 and BRM, both of which have been known to be crucial for skeletal muscle differentiation (Fedorov et al., 2015; Vangamudi et al., 2015; Gerstenberger et al., 2016; Sharma et al., 2021). To specifically investigate the requirement of BRG1 and/or BRM in the regulation of the Wnt signaling pathway, we performed siRNA-mediated individual and dual knockdowns of BRG1 and BRM followed by RNA-sequencing. A reduction of BRG1 and/or BRM protein levels and interference in myoblast differentiation due to siRNA treatment was validated, with BRG1 knockdown having a greater effect on differentiation (Supplemental Figures 2A–B), as has been previously shown (Albini et al., 2015; Sharma et al., 2021). Transcripts identified by RNA-seq were mapped to the mouse genome (mm10). Genes that were differentially expressed in both replicates for each siRNA and timepoint are presented in Supplementary Table S2-Supplementary Table S3-Supplementary Table S3 and were further analyzed. Differential gene expression analysis identified 5354 (2741 upregulated, 2613 downregulated), 7,290 (3,658 upregulated, 3,632 downregulated) and 1,201 (536 upregulated and 665 downregulated) targets upon knockdown of both BRG1 and BRM), BRG1 only and BRM only, respectively (Supplementary Table S2C). PANTHER pathway analysis of the differentially expressed genes due to individual or dual knockdowns showed that Wnt signaling pathway was listed as the most-affected pathway for knockdown of both BRG1 and BRM and of BRG1 only, but not for knockdown of BRM only (Figures 2A–C). The analysis of differentially expressed genes due to BRM knockdown identified integrin signaling pathways as a target of both BRG1 and BRM (Figures 2A–C). The findings are largely consistent with the analyses of the PFI-3 treated cells (Figure 1) and suggest that BRG1 and, in particular, the BRG1 bromodomain, promotes the ability of BRG1 to regulate the Wnt signaling pathway.

A heatmap of Wnt pathway-associated genes supports the idea that BRG1, but not BRM, regulates the expression of Wnt pathway and related genes. Expression of these genes were significantly altered in cells where BRG1 and BRM or BRG1 only were knocked down, whereas knockdown of BRM alone had minimal effects on gene expression (Figure 2D). Volcano plots of also demonstrate that knockdown of BRG1 or knockdown of both BRG1 and BRM resulted in differential expression of Wnt-related genes whereas these genes were not differentially expressed when BRM was knocked down (Supplementary Figure S3A). Validation of changes in the expression of selected Wnt pathway genes detected by RNA-seq was performed using quantitative RT-PCR (Figure 2E). Validation experiments also confirmed that expression of the genes encoding BRG1 and BRM were knocked down in the appropriate samples, as was expression of the gene encoding the early myogenic marker, myogenin. β-catenin mRNA levels were unaffected (Figure 2E). We also performed GO analyses of these datasets (Supplementary Figure S3B). The results indicate roles for BRG1 and BRM in skeletal muscle formation and function and are entirely consistent with prior evaluations of BRG1 and BRM knockdown in proliferating and differentiating myoblasts (Simone et al., 2004; De La Serna et al., 2006; Ohkawa et al., 2007; Forcales et al., 2012; Albini et al., 2015; Nasipak et al., 2015).

### β-catenin interacts with BRG1

**FIGURE 3:**
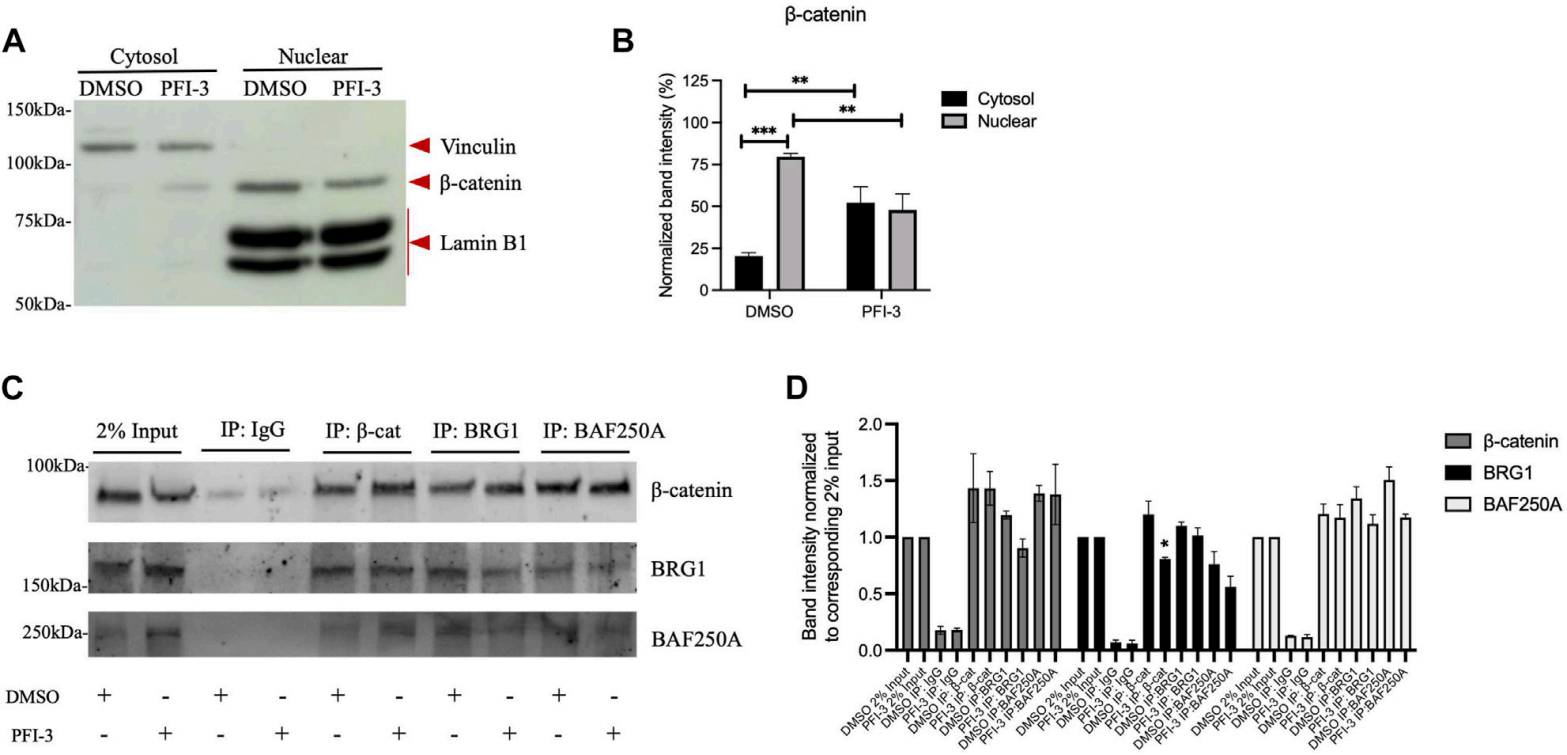
β-catenin interacts with BRG1 and the BAF250A subunit of mSWI/SNF enzymes. **(A)** Representative Western blot for C2C12 myoblasts treated with DMSO or PFI-3 and differentiated for 48 h were separated into cytosolic and nuclear fractions and immunoblotted for a cytosolic marker (Vinculin), a nuclear marker (Lamin B1) and β-catenin. **(B)** Quantification of the data in **(A)** showing the average values for 3 independent experiments ± standard deviation. **(C)** The nuclear fraction was subjected to co-IP using antibodies against β-catenin, BRG1 and BAF250A. Immunoblots show 2% input (lanes 1-2) and co-immunoprecipitated material for IgG (lanes 3-4), β-catenin (lanes 5-6), BRG1 (lanes 7-8), BAF250A (lanes 9-10) probed for β-catenin, BRG1, and BAF250A. **(D)** Quantification of co-immunoprecipitation experiments from 3 independent replicates. Data are presented as the mean ± standard deviation. For panels **(B)** and **(D)**, *p < 0.05, **p < 0.01, and ***p < 0.005 by Student’s t-test.

Although mSWI/SF enzymes remodel chromatin structure, they often physically interact with sequence-specific DNA-binding transcription factors (Pedersen et al., 2001; Kadam and Emerson, 2003; Salma et al., 2004; Seo et al., 2005; Eroglu et al., 2006; Hodges et al., 2016) to target to specific genomic regions. Given the indication that BRG1 mediates the expression of Wnt signaling pathway components (Figure 2), we therefore looked for interaction between BRG1 and BAF250A, a subunit of mSWI/SNF enzymes that is specific to the BAF subfamily of mSWI/SNF complexes (Kimelman and Xu, 2006), and β-catenin, which promotes Wnt target gene expression when translocated to the nucleus. Using C2C12 myoblasts differentiated for 48 h in the presence of DMSO or PFI-3, we fractionated whole cell extract into cytosolic and nuclear fractions (Figure 3A). β-catenin was detected in both cytosolic and nuclear fractions, in agreement with prior studies (Kimelman and Xu, 2006). Interestingly, there was less nuclear β-catenin and more cytoplasmic β-catenin in cells treated with PFI-3 as compared to the DMSO treated control cells (Figures 3A,B), suggesting decreased nuclear transport or increased nuclear export of β-catenin in PFI-3 treated differentiating C2C12 myoblasts. Using the nuclear fractions, co-immunoprecipitation experiments were performed for β-catenin, BRG1 and BAF250A. Immunoblots probed for β-catenin and BRG1 show interaction between these three proteins (Figure 3C). Quantification of three independent replicates of this experiment was performed (Figure 3D). Although we observed a reduction in the amount of BRG1 co-immunoprecipitated by β-catenin (Figures 3D – center) and a reduction in the amount of β-catenin co-immunoprecipitated by BRG1 (Figure 3D-left), only the former difference was statistically significant. Additionally, we note that there was no difference in the amount of co-immunoprecipitated protein in the analogous reciprocal experiment using β-catenin and BAF250A. We suggest that the reduction in nuclear β-catenin in PFI-3 treated cells is not reproduced in the co-immunoprecipitation experiments due to inefficiencies in the method itself that prevent qualitative immunoprecipitation of all of the nuclear protein. In summary, these results indicate that BRG1 and at least one other mSWI/SNF protein form a complex with β-catenin in the nucleus, suggesting a mechanism for the involvement of the mSWI/SNF complex in the expression of Wnt pathway genes.

### Rescue of the Wnt signaling pathway can restore myoblast differentiation in the presence of the PFI-3 bromodomain inhibitor or BRG1 knockdown

If inhibition of the BRG1 bromodomain or a deficiency in cellular BRG1 levels contributes to myoblast differentiation via regulation of Wnt signaling pathway components, agents that stabilize β-catenin may permit differentiation even in the presence of PFI-3 or BRG1 knockdown. Lithium chloride has been historically used as a Wnt pathway agonist for this purpose to understand the role of Wnt signaling pathway, as it inhibits GSK3β activity and therefore stabilizes β-catenin (Stambolic et al., 1996). A selective pharmacological inhibitor against GSK3β, CHIR99021, also stabilizes β-catenin (Ying et al., 2008) and so was used here to provide an additional test of the hypothesis.

**FIGURE 4:**
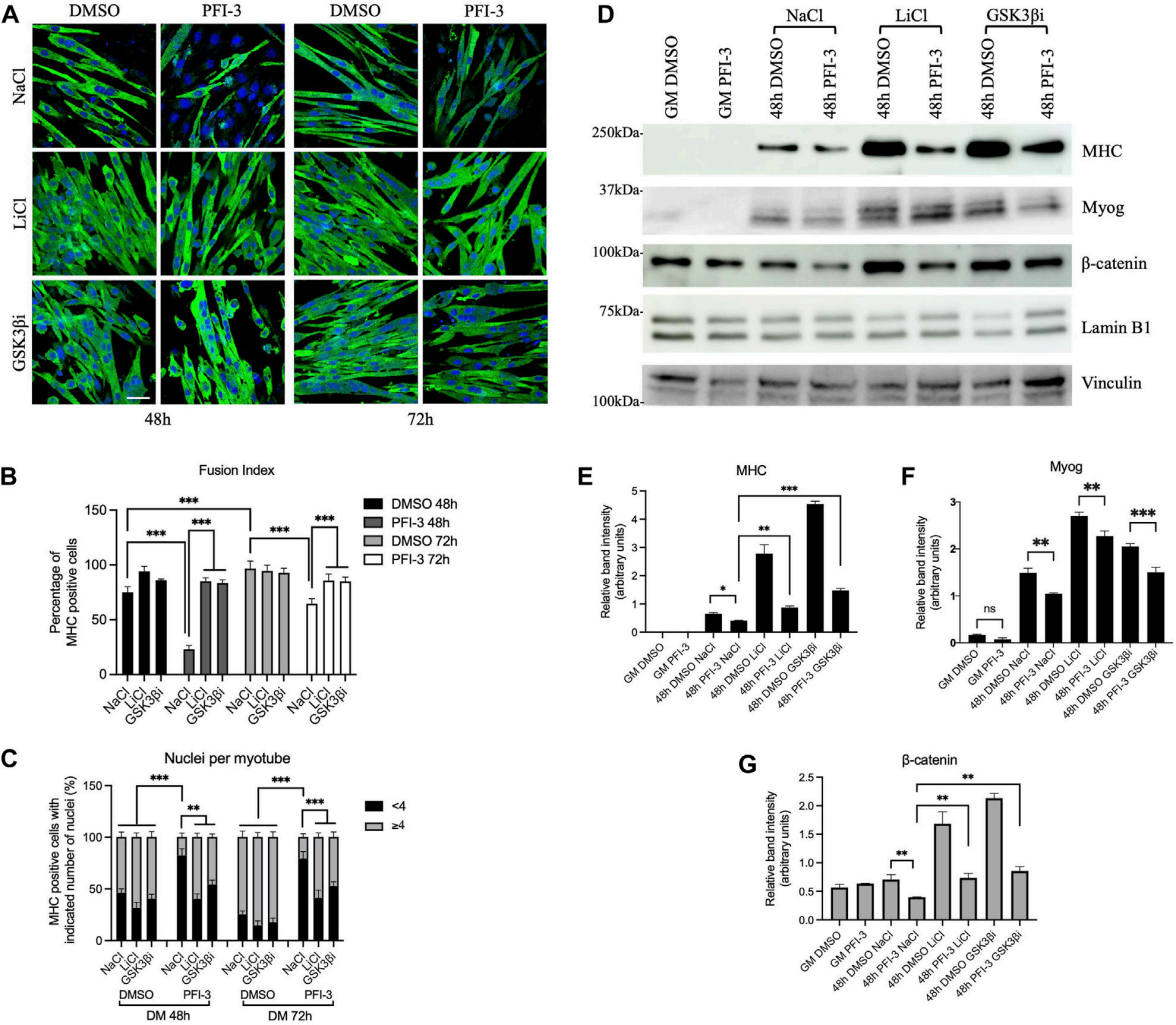
Rescue of Wnt signaling by β-catenin stabilization can restore myoblast differentiation in the presence of the PFI-3 bromodomain inhibitor. **(A)** Confocal images of 48 h differentiated C2C12 myotubes treated with DMSO or PFI-3. Both sample sets were subjected to GSK3β inhibition using LiCl or CHIR99021, a specific GSK3β inhibitor (GSK3βi), as described in the methods and immunostained for myosin heavy chain (FITC, green) and nuclei (DAPI, blue). NaCl was used as a treatment control. Scale:10 μm. **(B)** Fusion index and **(C)** nuclei per myotube were quantified from **(A)**. n > 150 nuclei. **(D)** Representative western blots showing expression of myosin heavy chain (MHC), myogenin (Myog), and β-catenin in 48 h differentiated C2C12 myotubes harvested after the indicated treatments. Lamin B1 was used as a loading control for the MHC and β-catenin blots; vinculin was used as the loading control for the myogenin blots. **(E–G)**. Bar plots showing band intensities for the proteins probed in **(D)** quantified using ImageJ (NIH). ns, not significant, *p < 0.05, **p < 0.01 and ***p < 0.005 by Student’s t-test. GM, proliferating cells in growth media.

As shown previously (Sharma et al., 2021), PFI-3 treatment inhibited myoblast differentiation at 48 and 72 h post-differentiation as monitored by imaging (Figure 4A) and determination of both the fusion index and the number of nuclei per cell (Figures 4B,C). Addition of LiCl or the GSK3β inhibitor rescued myogenic differentiation qualitatively and quantitatively by the measures described (Figures 4A–C). In addition, cells were probed for myosin heavy chain (MHC) and myogenin protein levels; the results showed that both LiCl and the GSK3β inhibitor significantly elevated MHC levels in untreated cells and elevated MHC levels in PFI-3 treated cells to or in excess of the MHC levels observed in the control cells (Figures 4D,E). Myogenin protein levels were also rescued (Figures 4D,F), though we note that PFI-3 had a smaller effect on myogenin expression than on MHC expression, consistent with prior observations (Sharma et al., 2021). Western blot analysis also confirmed that β-catenin levels were stabilized in the LiCl and GSK3β inhibitor-treated cells (Figures 4D,F). The results indicate that stabilizing β-catenin levels to rescue the Wnt signaling pathway can restore the ability of myoblasts to differentiate even in the presence of a BRG1/BRM bromodomain inhibitor.

**FIGURE 5:**
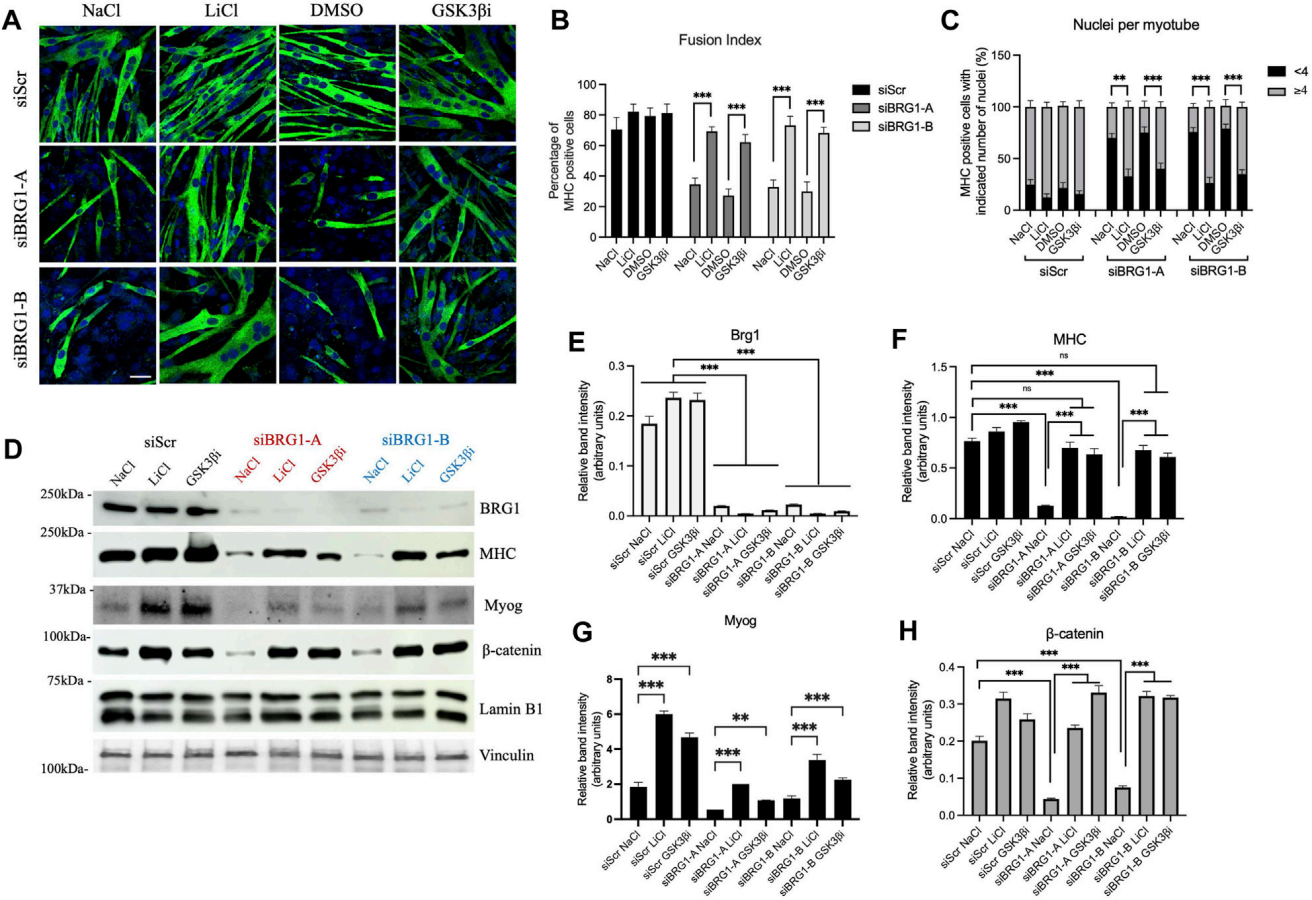
Rescue of Wnt signaling by β-catenin stabilization can restore myoblast differentiation under BRG1 knockdown conditions. **(A)** Confocal images of 48 h differentiated C2C12 myotubes knocked down for BRG1 using two different siRNAs (siBRG1-A, siBRG1-B) or a scrambled sequence siRNA (siScr) under the indicated conditions. Differentiating myoblasts were treated with LiCl or CHIR99021 (GSK3βi) as described in the methods and immunostained for myosin heavy chain (FITC, green) and nuclei (DAPI, blue). NaCl and DMSO were used as controls. Scale:10 μm. **(B)** Fusion index and **(C)** nuclei per myotube were calculated from **(A)** as described in methods. n > 150 nuclei. **(D)** Representative western blots showing expression of BRG1, myosin heavy chain (MHC), myogenin (Myog), and β-catenin in 48 h differentiated C2C12 myotubes harvested after treatments as described in **(A)**. Lamin B1 was used as the loading control for the MHC and β-catenin blots; vinculin was used as the loading control for the myogenin blots. **(E–H)** Bar plots showing quantification of band intensity for each of the probed proteins in the western blots shown in **(D)**. Data are presented as the mean ± standard deviation from three independent experiments. Quantification was performed using ImageJ, (NIH). ns, not significant, *p < 0.05, **p < 0.01 and ***p < 0.005 by Student’s t-test.

Similar experiments were performed using cells subjected to knockdown of BRG1. Two different siRNAs that target BRG1 were used (Supplementary Figure S4A). Knockdown of BRG1 inhibited myoblast differentiation as measured by imaging, fusion index, and number of nuclei per myotube (Figures 5A–C). Treatment with either LiCl or the GSK3β inhibitor restored differentiation as measured by all three assays (Figures 5A–C). Western blot analysis confirmed knockdown of BRG1 (Figures 5D,E) and showed that treatment with LiCl or the GSK3β inhibitor restored MHC and myogenin expression, though the extent of myogenin rescue was not as great and did not restore wildtype levels of myogenin to the treated cells (Figure 5D, Figures 5F-G). β-catenin levels were completely rescued from BRG1 knockdown by both Wnt pathway modulators (Figures 5D,H). These results confirm the previous results with PFI-3 (Figure 4) and demonstrate the importance of BRG1 in the regulation of the Wnt signaling pathway.

### BRG1 binds to Wnt pathway gene promoters

**FIGURE 6:**
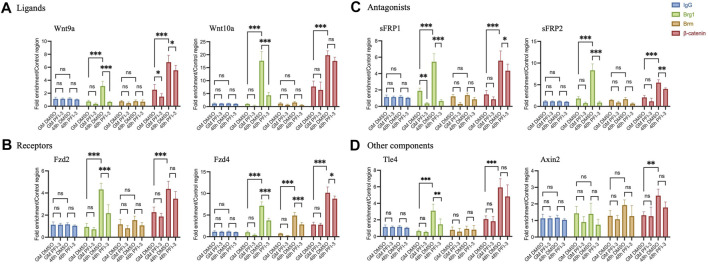
Chromatin immunoprecipitation assays show binding of BRG1, BRM, and β-catenin on gene promoters of Wnt pathway components- **(A)** Ligands- *Wnt9a* and *Wnt10a*, **(B)** Receptors- *Fzd2* and *Fzd4*, **(C)** Antagonists-*sFRP1* and *sFRP2*, **(D)** other components- *Axin*2 and transcription factor *Tle4*. Values were normalized to pulldowns with IgG and a genomic locus amplified by qRT-PCR as a negative internal control. Data are presented as the mean ± standard deviation from three independent experiments. ns, not significant, *p < 0.05, **p < 0.01 and ***p < 0.005 by Student’s t-test.

We asked whether BRG1 or BRM binds to Wnt pathway gene promoters to determine if they likely act in a direct manner on the regulatory sequences of these genes. We examined two ligand gene promoters, two receptor gene promoters, two antagonist gene promoters and two other gene promoters (Figures 6A–D). Proliferating myoblasts and myoblasts differentiated for 48 h were analyzed by chromatin immunoprecipitation (ChIP) under control conditions and in the presence of the PFI-3 bromodomain inhibitor. IgG pulldowns (Figures 6A–D) and amplification of unrelated genomic sequence (Supplementary Figure S4B) were performed as ChIP controls. BRG1 did not bind or bound at minimal levels to all promoters in proliferating myoblasts, and binding increased during differentiation at all of the promoters except for that controlling the *Axin2* gene. Binding was significantly impaired in the presence of the PFI-3 bromodomain inhibitor, thereby identifying the bromodomain as a contributor to the ability of the ATPase to interact with the chromatin at these promoter sequences. There was no indication of interaction of BRM with any of these sequences except at the *Fzd4* promoter, where it bound in a differentiation-specific and PFI-3 sensitive manner. This agrees with *Fzd4* transcript expression levels as seen in Figure 1D, indicating that *Fzd4* might be co-regulated by BRG1 and BRM. We conclude that BRG1 interacts with the regulatory sequences of most Wnt pathway genes.

We subsequently examined β-catenin binding to Wnt pathway gene promoters. β-catenin binding occurred in a differentiation-specific manner in PFI-3 treated and control cells at all of the tested loci (Figures 6A–D). Although a statistically significant decrease in β-catenin was observed at four of the eight promoters tested, the decrease in each case was 30% or less. We interpret this finding to suggest that β-catenin binding is not drastically compromised at Wnt pathway genes in differentiating myoblasts treated with the PFI-3 bromodomain inhibitor. This might be due to the effect of PFI-3 on β-catenin protein expression as seen in Figure 1E, however, further experimentation will be required to investigate the direct correlation of global β-catenin protein levels with its chromatin binding.

## Discussion

Skeletal muscle differentiation is regulated by a wide range of signaling pathways. Using the P19 trans-differentiation model, Petropoulos et al. (Petropoulos and Skerjanc, 2002) showed that β-catenin and the canonical Wnt pathway is essential and sufficient for skeletal myogenesis. The non-canonical pathway has been shown to contribute to satellite cell quiescence and control of mechanoproperties, which can be key during proliferation and myofiber regeneration upon injury (Eliazer et al., 2019). In this study, we investigated the role of BRG1-and BRM-containing mSWI/SNF complexes in the regulation of the canonical Wnt signaling pathway during myogenesis. Our data demonstrate that inhibition of BRG1 and BRM bromodomain function using a specific inhibitor results in dysregulated expression of Wnt pathway genes. Perturbation of endogenous gene expression of BRG1 and BRM using an siRNA-based approach helped us to further dissect that BRG1, and not BRM, contributes to transcriptional output of Wnt pathway components and Wnt target genes. We report that expression of Wnt pathway component genes is altered upon BRG1 knockdown or inhibition. The work adds regulation of expression of the Wnt pathway genes as a functional distinction between the BRG1 and BRM ATPases during myoblast differentiation.

A link between BRG1 and Wnt signaling was reported over 20 years ago, when it was determined that BRG1 and β-catenin interact to activate target genes (Barker et al., 2001). Mouse modeling identified BRG1 as an activator of Wnt signaling and demonstrated that pharmacological stabilization of β-catenin rescued target gene expression and proper vascular development (Griffin et al., 2011). Subsequent studies identified BRG1 as a co-activator of β-catenin in other developmental functions, including liver regeneration and the midblastula transition (Wagner et al., 2017). Our current study shows that cooperativity between BRG1 and β-catenin mediated regulation of Wnt signaling extends to myoblast differentiation.

Pharmacological stabilization of cellular β-catenin protein levels activated downstream Wnt signaling which, in turn, rescued the muscle differentiation phenotype and myogenic gene expression even upon BRG1 inhibition or knockdown. This indicates BRG1 control of Wnt signaling is upstream of β-catenin-dependent signaling. Chromatin immunoprecipitation assays showed binding of BRG1 on the regulatory regions of Wnt pathway genes in a differentiation-induced manner, and co-immunoprecipitation experiments showed that BRG1 and another mSWI/SNF subunit, BAF250A, interacted with the β-catenin effector of Wnt gene expression in nuclear extracts. These findings support a model where BRG1-based mSWI/SNF enzymes promote the appropriate regulation of Wnt pathway component genes and cooperate with β-catenin in the activation of Wnt signaling via direct stimulation of Wnt pathway genes.

Inhibition of BRG1 bromodomain function using PFI-3 was previously shown to inhibit myogenesis and to affect the recruitment of BRG1 to myogenic gene promoters and enhancers and the recruitment of BRM to the promoters of cell cycle regulators important in muscle differentiation (Sharma et al., 2021). BRG1 recruitment to promoters of Wnt pathway genes was also inhibited in the presence of PFI-3. This provides mechanistic insight into BRG1-dependent regulation of Wnt signaling during skeletal myogenesis by indicating a role for the BRG1 bromodomain, which is known to interact with acetylated histones (Shen et al., 2007; Singh et al., 2007), a hallmark of actively transcribed genes. The results suggest optimal BRG1 binding requires interaction with acetylated chromatin and at least partially explains how BRG1 contributes to Wnt pathway gene expression via a role in transcriptional activation. BRG1 function during regulation of Wnt pathway components is, however, more complex, as there is bromodomain-dependent repression of the genes encoding the Wnt antagonists Sfrp1 and Sfrp2. Further work on this topic would require examination of the histone acetylation and other post-translational marks on these and other genes showing BRG1-dependent repression. In other systems, studies have shown that BRG1 can epigenetically regulate the Wnt pathway in regenerating liver after partial hepatectomy by recruiting the histone demethylase KDM4 to β-catenin target genes (Li et al., 2019). Another study performed in liver tumor inducing cells showed direct binding of BRG1 at *Fzd6* promoter during tumor inducing cell self-renewal (Chen et al., 2018), thereby providing additional support for BRG1 as a transcriptional regulator of genes encoding components of the Wnt pathway.

β-catenin is a key effector component of the canonical Wnt pathway. Interestingly, we saw no change in expression of β-catenin at the transcript level due to BRG1 knockdown or PFI-3 induced inhibition (Figure 1D; 2E). However, there was decreased protein expression, which argues for regulation at the post-transcriptional level (Figure 1E; Figures 4FD,G; Figures 5D,H). Thus, we provide evidence of a dual role for BRG1 in regulating the Wnt pathway; Wnt pathway component gene expression is regulated at the level of transcription while β-catenin gene expression is regulated post-transcriptionally. We do not know whether this post-transcriptional regulation involves mechanisms relating to the stability, splicing, or translational potential of the β-catenin mRNA or whether BRG1 and mSWI/SNF enzymes indirectly affect the expression of proteins involved in post-transcriptional processes. An intriguing possibility is that members of the β-catenin degradation complex are targeted. Review of differentially regulated genes induced by 48 h of PFI-3 treatment (Sharma et al., 2021) or by knockdown of BRG1 or both BRG1 and BRM, but not by BRM knockdown alone (Supplementary Table S2), showed downregulation of multiple members of this complex. Thus, a possible mechanism to explain post-transcriptional regulation of β-catenin protein levels may involve the transcriptional regulation of components of the β-catenin degradation complex by BRG1.

Axin2 is a component of the β-catenin-degradation complex and is also a downstream Wnt target (Novak and Dedhar, 1999; Nusse and Clevers, 2017). We observed some intriguing but unexpected results related to its transcript expression when differentiating C2C12 cells were subjected to knockdown or inhibition of BRG1 and BRM. In case of dual knockdown of BRG1 and BRM, there was no change in *Axin*2 expression (Figure 2E). There was, however, a decrease in expression when BRG1 was knocked down and a reverse effect when BRM was depleted. A possible explanation could be a balanced regulation of *Axin2* expression by BRG1-and BRM-containing mSWI/SNF complexes that is tilted one way or the other in the absence of one of the ATPases. Inhibition of bromodomain function by PFI-3 resulted in a modest reduction of *Axin2* mRNA expression, suggesting that inhibiting BRG1 bromodomain function had a greater impact than simultaneous inhibition of BRM bromodomain function. The reason for *Axin2* sensitivity to BRM while most of the other Wnt signaling pathway components did not show regulation by BRM is not known. The difference in transcript output could possibly be a result of variable mode of BRG1/BRM functional manipulation. We note that neither BRG1 nor BRM bound to *Axin2* sequences even though β-catenin binding was detected (Figure 6), suggesting that BRG1 and BRM-mediated regulation occurs via an indirect mechanism. However, we did not extensively survey the entire *Axin2* locus. An alternative hypothesis is that BRG1 and/or BRM bind elsewhere on the *Axin2* locus.

It is worth noting that other signaling pathways, such as integrin and Rho GTPase signaling, are also altered due to BRG1 knockdown or inhibition of bromodomain function (Figures 1A,B; Figures 2A,B). The role of extracellular matrix-dependent signaling pathways is linked to the mechano-contractile nature of the skeletal muscle tissue (Eliazer et al., 2019). The non-canonical Wnt pathway consists of a Rho GTPase-dependent pathway that contributes to planar cell polarity and a phospholipase C-dependent pathway that responds to fluctuations in cellular Ca^2+^ levels (Miller et al., 1999; Bentzinger et al., 2012). The expression of Wnt pathway genes as seen in the heatmaps (Figure 1C; Figure 2C) also depicts altered expression of Ca2+-calmodulin kinases and NFAT transcription factors that belong to the non-canonical branch. Thus, the non-canonical Wnt pathway might also be altered due to BRG1 knockdown or bromodomain inhibition. Further experiments would be required to explore BRG1-mediated regulation of the non-canonical Wnt pathway.

It has long been appreciated that mSWI/SNF enzymes contribute to myogenesis, but prior work has largely focused on the functions of these enzymes in the regulation of myogenic genes required for differentiation and maintenance of the muscle phenotype and of genes involved in cell cycle control. Our findings indicate a previously unappreciated role for BRG1 and the mSWI/SNF enzymes in the regulation of the Wnt signaling pathway during skeletal muscle differentiation. This function is largely independent of the homologous BRM ATPase. mSWI/SNF enzymes containing BRG1 transcriptionally regulate Wnt pathway genes via binding to sequences upstream of these genes and through a post-transcriptional mechanism that impacts the protein levels of the β-catenin regulatory protein that is central to Wnt signaling. The integration of mSWI/SNF function in promoting myogenic differentiation through multiple mechanisms reflects the complexity of differentiation signaling and the important roles for these chromatin remodeling enzymes.

## Data Availability

The datasets presented in this study can be found in online repositories. The names of the repository/repositories and accession number(s) can be found below: https://www.ncbi.nlm.nih.gov/geo/query/acc.cgi?acc=GSE196283, GSE196283.
