# Supplementary material for: Regulation of the Wnt signaling pathway during myogenesis by the mammalian SWI/SNF ATPase BRG1
Source: Front Cell Dev Biol. 2023 Jul 7;11:1160227. doi: 10.3389/fcell.2023.1160227 (PMC10360407; doi:10.3389/fcell.2023.1160227)
Supplement: Supplementary file 3 [file DataSheet1.pdf]

Supplemental Figure 1

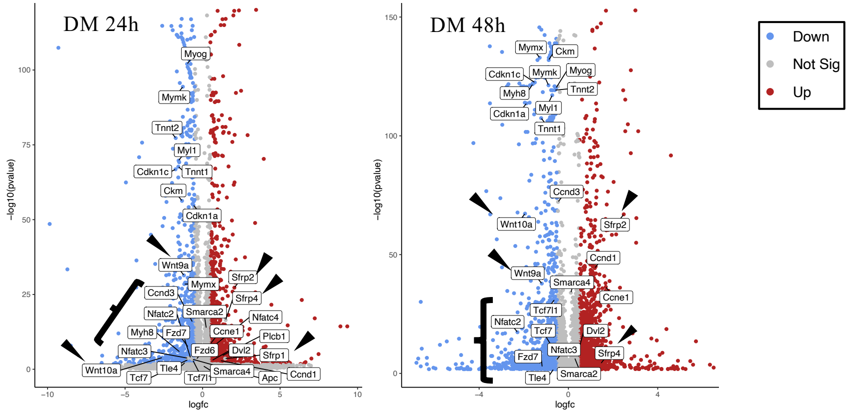


**Supplemental Figure 1.** Volcano plots for differentially expressed genes identified from RNA-sequencing of DMSO- or PFI-3-treated C2C12 myoblasts differentiated for 24h and 48h. Gene symbols for some myogenic and cell-cycle related genes are highlighted in rectangular boxes. Genes from Wnt-signaling pathway are marked with black arrows/brackets. DM, differentiation media; logfc, log fold-change.

Supplemental Figure 2

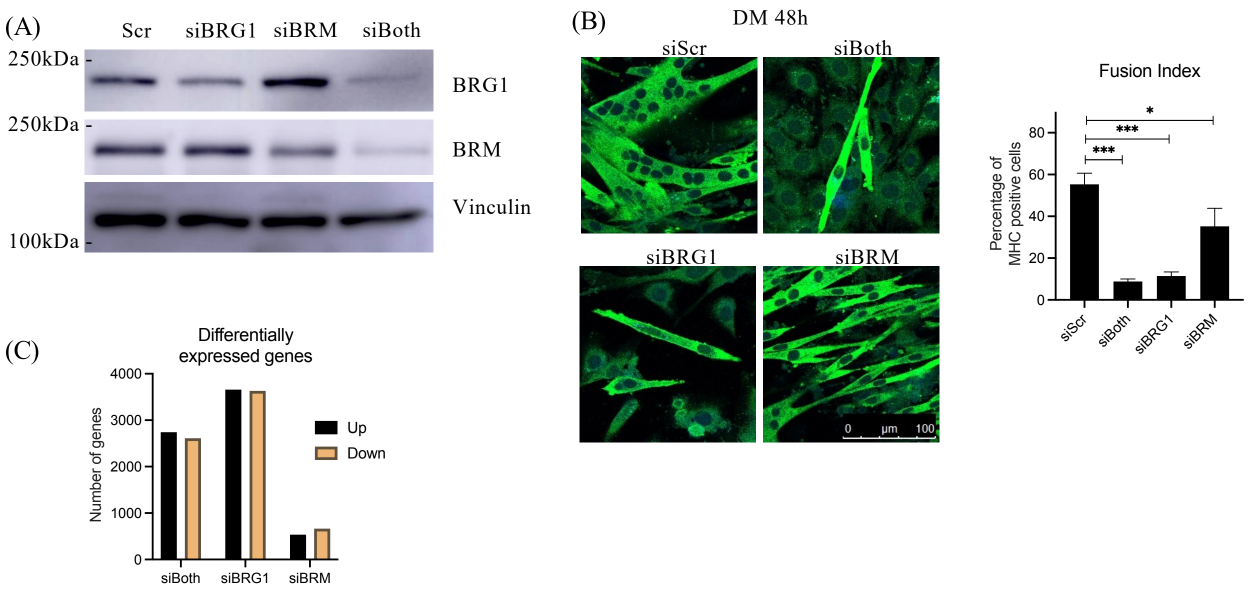


**Supplemental Figure 2.** (A) Representative western blots for expression of BRG1 and BRM in 48h differentiated C2C12 myotubes treated with siRNA against non-targeting scrambled (Scr), BRG1, BRM or both. Vinculin was used as a loading control. (B) Representative confocal images of 48h differentiated C2C12 myotubes as described in (A) and immunostained for myosin heavy chain (FITC, green). Quantification of fusion index is provided in the adjoining bar graph. (C) Number of differentially expressed genes in 48h differentiated C2C12 myoblasts described in (A) as determined by RNA-sequencing analysis. *$p<0.05$, ***$p<0.005$. DM, differentiation media.

Supplemental Figure 3

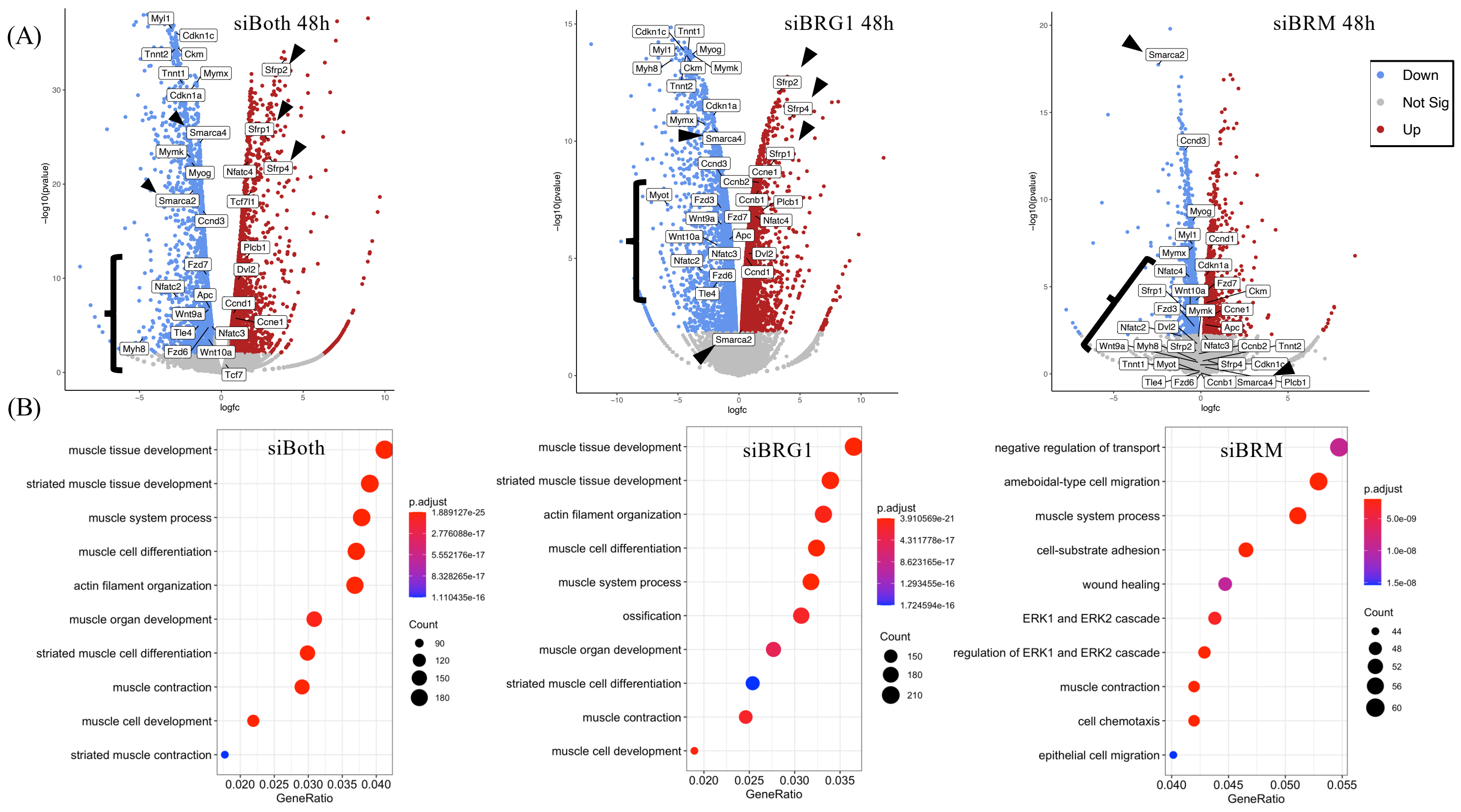


**Supplemental Figure 3.** (A) Volcano plots for differentially expressed genes identified from RNA-sequencing of 48h differentiated C2C12 myotubes treated with siRNA against BRG1, BRM or both. Differential gene expression is calculated relative to the corresponding control samples (siScr). Gene symbols for some myogenic and cell-cycle related genes are highlighted in rectangular boxes. Genes from Wnt-signaling pathway are marked with black arrows/brackets. (B) GO analysis of differentially expressed genes as described in (A). Top 10 categories related to biological processes are shown.

Supplemental Figure 4

(A)

(B)

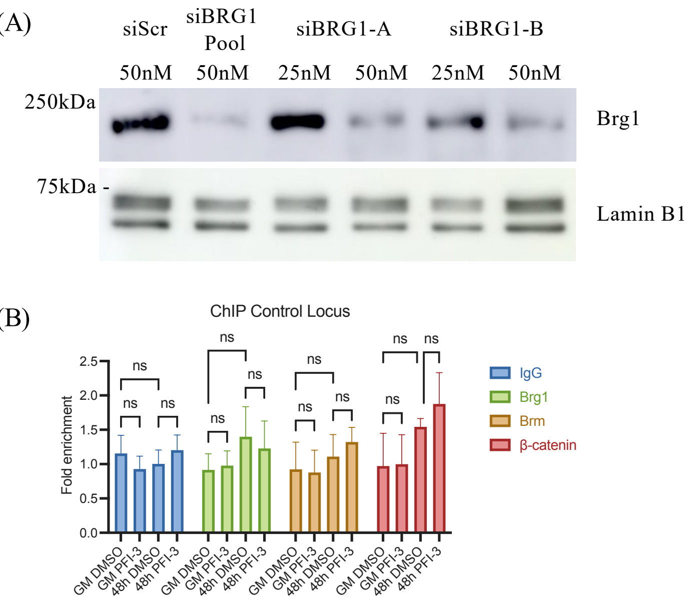


**Supplemental Figure 4.** (A) Representative western blot showing expression of BRG1 in 48h differentiated C2C12 myoblasts treated with siRNA pool against BRG1 (siBRG1 pool) or two individual siRNAs (siBRG1-A and siBRG1-B) at the indicated concentrations. Lamin was used as a loading control. (B) Plot showing amplification of the control genomic locus using DNA obtained from C2C12 myoblasts assayed for chromatin immunoprecipitation with antibodies against control IgG, BRG1, BRM, and β-catenin.

## LEGENDS FOR SUPPLEMENTAL TABLES

**Supplemental Table 1.** List of primers used for qRT-PCR and ChIP assays.
**Supplemental Table 2.** List of differentially expressed genes (gene symbols with Entrez IDs) identified from RNA-sequencing of 48h differentiated C2C12 myotubes treated with siRNA against non-targeting scrambled (Scr), BRG1, BRM or both BRG1 and BRM.
**Supplemental Table 3.** List of differentially expressed genes from Supplemental Table 2 with detailed fold change in expression and p-values
